# Evaluation of proteolytic activity and serine proteases distribution in plasma from patients with bladder cancer

**DOI:** 10.3389/fmed.2023.1276882

**Published:** 2023-11-15

**Authors:** Tatyana Synelnyk, Tetiana Vovk, Tetiana Halenova, Valentyn Tytarenko, Nataliia Raksha, Olexii Savchuk, Tetyana Falalyeyeva, Liudmyla Ostapchenko, Pavel Yakovlev, Marko Kozyk, Dominic Thorley, Kateryna Strubchevska

**Affiliations:** ^1^Taras Shevchenko National University of Kyiv, Kyiv, Ukraine; ^2^Department of Anatomy Bogomolets National Medical University, Kyiv, Ukraine; ^3^Feofaniya Clinical Hospital, Kiev, Ukraine; ^4^Corewell Health William Beaumont University Hospital, Royal Oak, MI, United States; ^5^Lake Erie College of Osteopathic Medicine (LECOM), Erie, PA, United States

**Keywords:** bladder cancer, serine proteases, proteolysis, plasminogen, elastase, electrophoresis

## Abstract

**Background:**

Bladder cancer (BC) is an aggressive disease with a poor prognosis. A bladder tumor, like other malignant neoplasms, is characterized by the presence of both cancer cells and stromal cells which secrete cytokines, chemokines, growth factors, and proteolytic enzymes. One such class of proteolytic enzymes are serine proteases, which take part in the tumor microenvironment formation via supporting and contributing to tumor progression. This study aims to evaluate the proteolytic activity and serine protease contribution in plasma from BC patients.

**Methods:**

The research involved patients of Alexandrovsky city clinical hospital aged 52–76 with transitional cell carcinoma of the bladder. All examined patients were divided into five groups: the control group included conditionally healthy donors, while other patients were grouped according to their tumor stage (I, II, III and IV). Plasma plasminogen levels were determined by enzyme-linked immunosorbent assay, and the potential activity was measured by chromogenic plasminogen assay. Serine proteases fractions were obtained by the affinity chromatography method, and enzyme concentration in the selected fractions were determined by the Bradford method. Serine proteases distribution was investigated by electrophoresis in a polyacrylamide gel.

**Results:**

It was determined that the concentration, potential activity of plasminogen, and the total amount of serine proteases in plasma from BC patients were greater than the values of the corresponding indicators in healthy donors. This could be one of the factors contributing to increased proteolysis seen in the process of carcinogenesis. Plasminogen concentration in BC patients with stage IV disease; however, displayed a tendency to be reduced compared to earlier stages, and the potential activity of plasminogen was significantly lower in patients with stages III – IV BC. Futhermore, a tumor stage specific gradual decline in the serine protease plasma content was shown. The results of electrophoretic analysis established a significant diminishment in the percentage of high molecular weight components (under non-reducing conditions) and their complete disappearance (under reducing conditions) in plasma serine protease fractions from BC patients. A decline in the percentage of heavy and light plasmin chains in BC patients was also observed. Additionally, a rise in the degraded forms of plasminogen/plasmin content was seen in BC samples, as well as the presence of fractions corresponding to trypsin and NE (under non-reducing conditions) that were absent in the control samples.

**Conclusion:**

The results indicate significant changes in the proteolytic activity of plasma, from BC patients when compared to healthy controls, which is accompanied by alterations in serine protease distribution caused by tumor microenvironment pecularlities at the different stages of oncopathology.

## Introduction

Bladder cancer (BC) is an aggressive disease with a poor prognosis. It is the fourth most common oncological disorder in men (10% of all cancers) and the eighth most common in women (4%). The disease affects older people at an increased incidence – 9 out of 10 diagnoses occur in adults aged 55 and older (1).

Usually, the development of BC originates from the epithelium (urothelium), which lines the inner surface of the bladder. Therefore, bladder carcinoma, also called urothelial carcinoma, is the most frequent BC type (90% in Europe and the USA) (2). Approximately 60–75% of patients are diagnosed with an early stage of non-muscle invasive BC (NMIBC), which corresponds to stages Ta, Tis/CIS, and T1. Only about 25% of patients have muscle-invasive BC (MIBC) (stages T2–T4), including 5% BC patients with metastatic disease.

When NMIBC is diagnosed, patients have a good prognosis – the 5-year and 10-year survival rates of such patients are 88 and 86%, respectively. However, the disease is characterized by a high rate of recurrence (up to 80%), and progresses with MIBC phenotype formation in 10–30% of cases due to undetected tumours under cystoscopy, incomplete excision, or re-implantation of a tumor during transurethral resection of a bladder tumor (3, 4). MIBC is a more aggressive tumor with 5-year survival rate about 46% (stage III) or less than 15% (stage IV or metastases) (5). Thus, the high recurrence rate in combination with disease heterogeneity demonstrate the relevance of research into bladder carcinoma progression mechanisms, the development of alternative approaches to early BC detection, and the search for new markers useful for both assessing the prognosis and monitoring the patient’s condition during treatment (6–9).

The cancerous component of the tumor in combination with the extracellular matrix (ECM) and stromal cells create a unique medium that adapts to the needs of a growing tumor, allows it to develop, and promotes its malignancy (2, 10, 11). Both cancer and stromal cells secrete cytokines, chemokines, and growth factors that contribute to tumor progression and cause resistance to anticancer therapy. These cells also secrete proteases, which not only participate in ECM degradation and remodeling, but also affect the expression, processing, and bioavailability of cytokines, chemokines, and growth factors, and therefore, depending on a number of conditions, can have either a tumor-stimulating or a tumor-suppressive effect (12, 13).

All together, the complex network of proteases, their substrates, and their inhibitors, interconnected through a number of links, is called a degradome (14). As part of cancer pathology, proteases released from cancer and stromal cells form a specific cancer degradome which is involved in all stages of cancer progression, such as the initiation of epithelial-mesenchymal transition, tumor growth, immune cell recruitment, resistance to apoptotic signals, evasion of the immune system, angiogenesis, invasion, and metastasis (13, 15).

Human proteolytic enzymes are represented by at least 580 proteases. Based on their catalytic mechanism, they are classified into metallo-, serine, cysteine, threonine and aspartic proteases (15). Serine proteases account for more than a third of all known proteolytic enzymes. They are involved in a variety of physiological processes, and some of them also play an important role in carcinogenesis. In particular, plasminogen, plasmin, and plasminogen activation system (PAS) components such as urokinase-type and tissue-type plasminogen activators (uPA and tPA, respectively), directly participate in ECM remodeling as well as proteolytically activate matrix metalloproteinases (MMPs) and some grouth factors, and therefore influence on the angiogenesis, invasion, and metastasis processes (16, 17).

Considering the above, this study aimed to evaluate the proteolytic activity and serine proteases distribution in plasma from BC patients.

## Materials and methods

### Participants and study design

This study involved 40 male BC patients of Alexandrovsky city clinical hospital of Kyiv, Ukraine, aged 52–76 years. Only men were included in the study group, because according to statistics, ВС incidence is significantly higher in males than females (1); therefore, the study referred to indicators determined specifically in men with ВС. All patients had transitional cell carcinoma of the bladder and underwent standard preoperative check-up, including general blood analysis, urine analysis, blood chemistry, and blood immunogram. Patients also underwent computed tomography of the pelvis, abdomen, and chest cavity with contrast enhancement to determine the depth of the tumorous lesion in the bladder, and the presence of regional or distant metastases. Concomitant diseases, participation of patients in other researche, as well as treatment that could affect the studied indicators values were used as exclusion criteria. To characterize the tumor stage, TNM clinical classification of the American Joint Committee on Cancer, 8^th^ edition, was used. All examined patients were divided into the following groups: Stage I (*n* = 10); Stage II (*n* = 10); Stage III (*n* = 10); Stage IV (*n* = 10). The control group included 10 conditionally healthy donors (men) of respective age without clinical signs of BC and a history of oncological disorders in the past. All healthy donors and patients (or their respective relatives) were informed about the clinical research protocol and have given written informed consent to participate in the study. The research was performed in accordance with the principles outlined in the World Medical Association’s Declaration of Helsinki, fully met the ethical and moral requirements of the current provisions of the Ministry of Health of Ukraine, and was approved by the Ethics Committee at the ESC “Institute of Biology and Medicine,” Kyiv, Ukraine (the protocol No 9, 13.11.2020).

### Sample collection and processing

The morning before the operation venous whole blood was collected from hospitalized patients and healthy donors into commercially available citrate-treated tubes. All patients fasted for 8 h before blood sampling. Plasma was obtained by citrated anticoagulated blood centrifugation at 2500 × g for 25 min.

### ELISA analysis

Plasma plasminogen levels were determined by enzyme-linked immunosorbent assay (ELISA) according to (18). Before use, blood plasma was diluted to 10 μg/mL with 50 mmoL/L Tris–HCl buffer (pH 7.4). Primary polyclonal antibodies against plasminogen (plasminogen (G-7): sc-376405; Santa Cruz Biotechnology, Dallas, TX, USA) and secondary antibodies conjugated with horseradish peroxidase (Goat Anti-Mouse IgG (H + L)-HRP Conjugate #1706516; Bio-Rad Laboratories, Inc., USA) were used. After the last washing of the plate with 50 mmoL/L Tris–HCl buffer (pH 7.4), substrate development was performed with chromogenic a mixture o-phenylenediamine (Sigma-Aldrich, Louis St, MO, USA) and H_2_O_2_ in 0.1 M sodium citrate buffer, pH 5.0. The reaction was terminated by the addition of 1 N H2SO4. The optical density of the samples was measured using a microplate reader (μQuantTM, BioTek Instruments, Inc., Winooski, VT, USA) at a wavelength of 492 nm (19). The relative content of plasminogen was expressed as a percentage, taking the value of this indicator in the control as 100%.

### Chromogenic plasminogen assay

Potential plasminogen activity was measured by streptokinase method based on plasminogen activation to plasmin by streptokinase; plasmin in turn cleaves a specific chromogenic substrate. The measured change in sample optical density is directly proportional to potential plasminogen activity (20). Potential plasminogen activity was determined using commercial reagents (RENAU, Kharkiv, Ukraine). Plasma was diluted 1:50 by 0.05 moL/L Tris–HCl buffer (pH 7.4). Streptokinase with activity of 50 IU was used. Chromogenic substrate S2251 was added at a final concentration of 3 mmoL/L. The optical density of the samples was measured at 405 nm using a microplate spectrophotometer (BioTek Instruments, Winooski, Vermont, USA). The relative potential plasminogen activity was expressed as a percentage, taking the value of the indicator as 100%.

### Affinity chromatography

The serine proteases fractions were isolated by affinity chromatography method with benzamidine-sepharose as a sorbent (21). A benzamidine-sepharose column was pre-equilibrated with 10 volumes of Tris–HCl buffer (10 mmoL/L, pH 8.0). To remove unbound material, the column was washed with 15 volumes of Tris–HCl buffer (10 mmoL/L, pH 8.0). For elution, a buffer solution with the following composition was prepared: 50 mmoL/L glycine-HCl and 1 moL/L NaCl (pH 3.0).

### Bradford method

Taking into account that the total protein concentration in the selected serine proteases fractions corresponds to the studied enzymes amount, their content in the fractions obtained from plasma was determined by the Bradford method according to the protocol (22) and expressed in milligrams per 1 mL.

### Disk-electrophoresis in a polyacrylamide gel

SDS-PAGE was carried out in 10% separating gel according to the Laemmli method (23). Electrophoresis was performed at 19 mA for stacking gel and 36 mA for separating gel. Samples after the affinity chromatography step were mixed with sample buffer (5 mmoL/L Tris–HCl (pH 8.8), 2% SDS, 5% sucrose, and 0.02% bromophenol blue) in the ratio of 1:1 (v/v). The samples were heated at +95°C for 40–50 s prior to loading in the gel. The total amount of proteins applied per well of gel was 20 μg. After separation, the gels were stained with 2.5% Coomassie brilliant blue R-250 in 10% (v/v) ethanol, 10% (v/v) acetic acid, and 15% (v/v) isopropanol for 30 min. Excess dye was removed from gels by boiling in a 2–8% acetic acid solution. The molecular weights of proteins were estimated using a protein calibration mixture (Bio-Rad Laboratories, Inc., Hercules, California, USA). To carry out electrophoresis under reducing conditions, β-mercaptoethanol was previously added to the samples. The electropherograms were analyzed using the TotalLab 2.04 program (TotalLab Ltd., UK).

### Statistical analysis

Data entry and analysis were performed using MS Excel (MS Office) and StatSoft Statistica ver.8.0 for Windows. After testing for normality (by Shapiro–Wilk), a one-way analysis of variance (ANOVA) was used to compare the means among different groups. Differences were statistically significant when *p* < 0.05.

## Results

Components of PAS, and plasmin which is generated by this system, play an important role in tumor development and progression. Therefore, the first stage of our research was to determine the content of plasminogen and its potential activity in plasma from patients with different BC stages.

**Figure 1:**
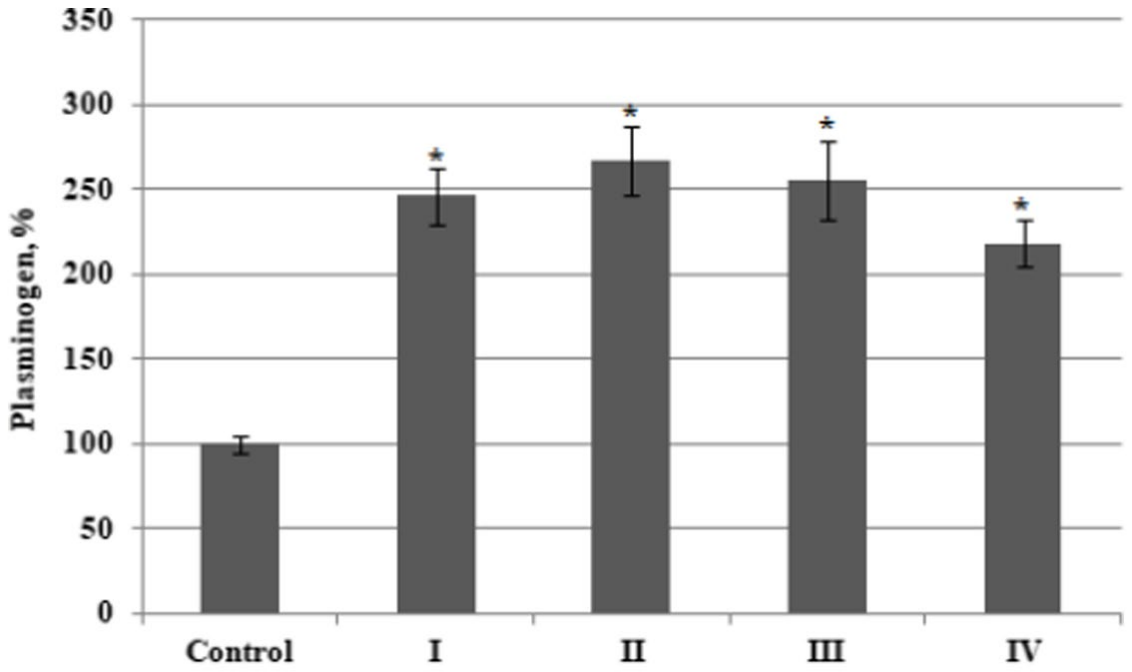
The relative content of plasminogen in plasma from bladder cancer patients with different (I – IV) stages of disease (*n* = 10 for each stage) and healthy control (Control, *n* = 10), percentage from control value. Data are presented as the mean ± SD. Statistical analysis based on the One-way ANOVA; *p ≤ 0.05, comparing to control group.

Our results showed that the plasminogen content increased by an average of 2.6 fold in plasma from patients with BC Stages I – III compared to the value of this parameter measured in plasma from healthy donors. This value also increased, although less significantly, by a factor of 2.2 in patients with stage 4 disease (Figure 1). No significant differences in plasminogen plasma content were found when comparing the samples between different BC stages.

**Figure 2:**
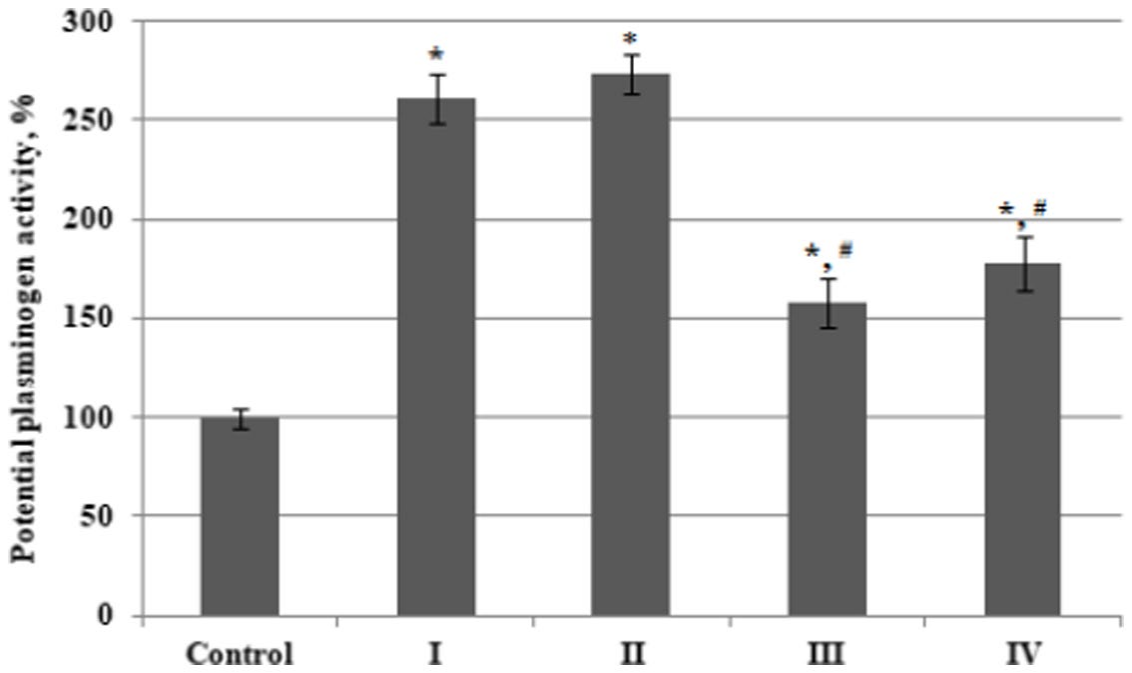
The relative potential activity of plasminogen in plasma of bladder cancer patients with different (I – IV) stages of disease (*n* = 10 for each stage) and healthy control (Control, *n* = 10), percentage from control value. Data are presented as the mean ± SD. Statistical analysis based on the One-way ANOVA; *p ≤ 0.05, comparing to control group and #*p* ≤ 0.05, comparing to stage I.

The potential activity of plasminogen, assessed using the streptokinase method, reached the maximum values in plasma from BC patients with Stages I and II (increased 2.6 and 2.7 fold from thevcontrol values) and increased less prominently in BC patients with stages III (1.6 times) and IV (1.8 times) cancer (Figure 2).

As a result, the values of relative plasminogen acticity in the third and fourth stage of disease were significantly lower than that in patients with stage 1 or stage 2 BC.

Such a decrease in the potential activity of plasminogen in the last stages of BC, with an almost invariably high content of this protein in the blood plasma, may be the result of a derangement of its activation and, accordingly, a decrease in the generation of plasmin due to certain changes in the activities of proteases involved in the transformation of plasminogen during tumor progression. To test this hypothesis, as well as to establish the peculiarity of blood plasma proteolytic activity in patients with different stages of BC, the qualitative and quantitative composition of plasma serine protease fractions was investigated.

**Figure 3:**
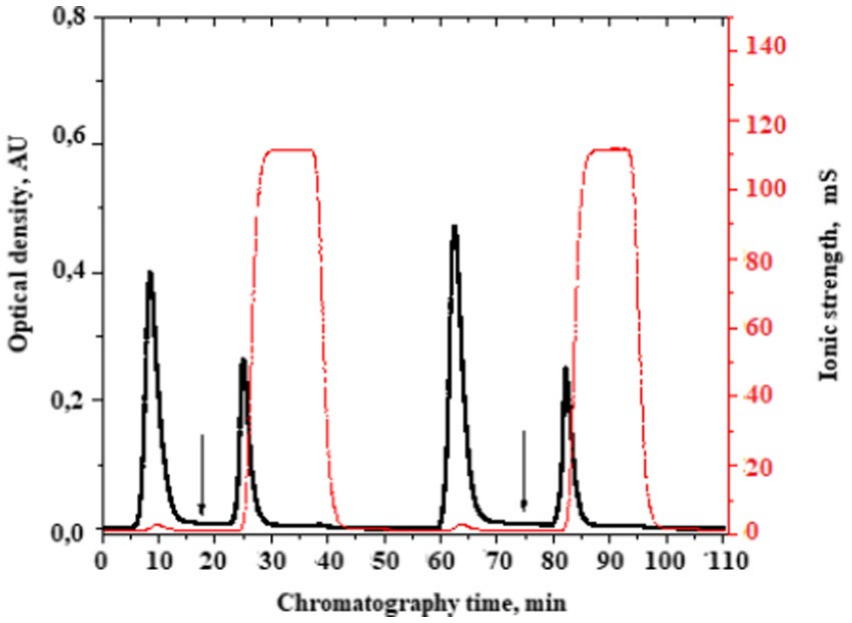
A typical chromatogram of the serine protease fraction isolation from the plasma of patients with bladder cancer. The moment of the serine protease fraction elution start is indicated by the arrow. Here, the buffer of 10 mmoL/L Tris–HCl, pH 8.0 was changed into a buffer of 50 mmoL/L glycine-HCl, pH 3.0 + 1 moL/L NaCl.

We performed separation by affinity chromatography on benzamidine-sepharose to isolate these fractions (Figure 3).

**Figure 4:**
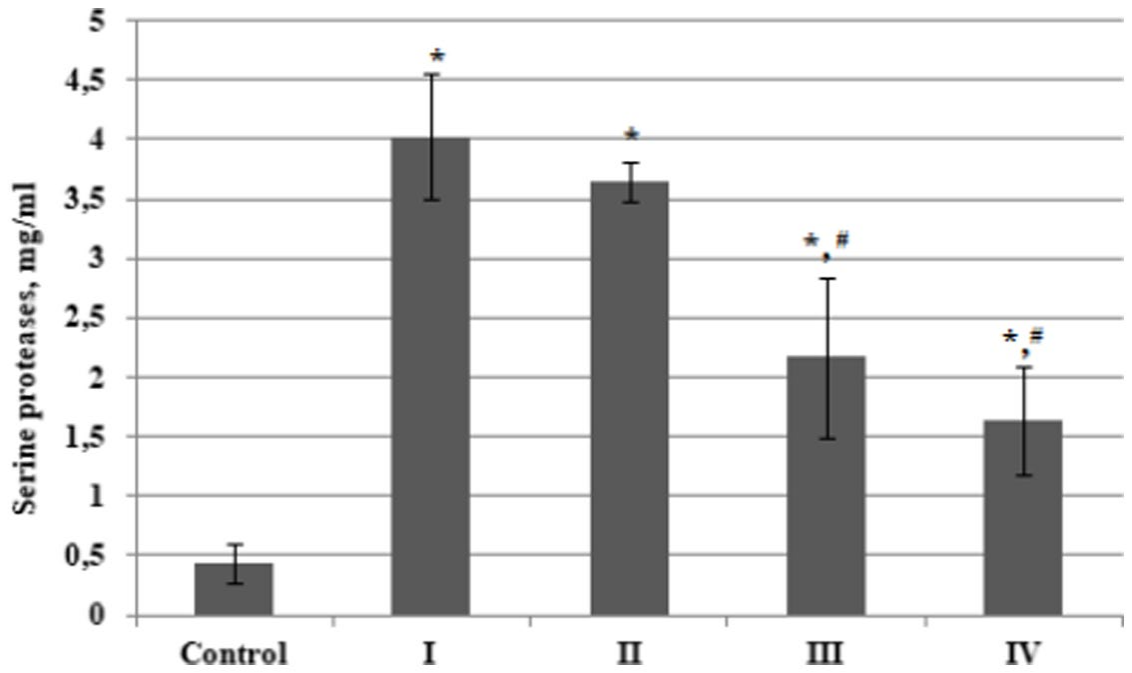
Plasma concentration of serine proteases in bladder cancer patients with different (I – IV) stages of disease (*n* = 10 for each stage) and healthy control (Control, *n* = 10), mg/ml. Data are presented as the mean ± SD. Statistical analysis based on the One-way ANOVA; *p ≤ 0.05, comparing to control group and #*p* ≤ 0.05, comparing to stage I.

In order to compare the total serine proteases content in plasma from BC patients with that from the control group, as well as to evaluate the values of this indicator in plasma from patients with different stages of oncopathology, we determined these enzymes content in the isolated fractions according to the Bradford method. It was established that the total content of serine proteases in plasma from patients with BC stage I was almost 9 times higher compared to the control group (Figure 4). There were significant differences in the values of this indicator at different stages of BC, but the total plasma serine proteases content in patients with oncopathology was always higher than in the control group. Moreover, we found a significant decline in this indicator level in the later stages of the disease; BC Stages III and IV.

Composition of isolated fractions were further analyzed by disk electrophoresis in a polyacrylamide gel in the presence of SDS in two variants – under reducing conditions (with β-mercaptoethanol addition) and under non-reducing conditions (without this compound). Gel electrophoresis under reducing conditions was used to study the oligomeric structure of proteins because reducing agents destroy disulfide bonds, which allows the protein molecule to be separated into individual monomers. The total amount of proteins in the serine protease fraction obtained from blood plasma of each experimental group was set up as 100%, and the level of a particular protein fraction was expressed as a percentage of the total value.

According to the results of electrophoresis carried out under non-reducing conditions, it was established that plasma serine protease fractions from both healthy people and patients with different stages of BC were divided into 5 bands which corresponded to the following molecular weight ranges: 20, 28, 45–75, 95, 105–220 kDa. In accordance with data already in the literature, these values are matched to trypsin (23.3 kDa), neutrophil elastase (28 kDa), plasmin (A-chain – 60 kDa, B-chain – 25 kDa), plasmin degraded forms and, plasmin complexes with inhibitors (Table 1).

**Table 1:** Protein composition of plasma serine protease fractions from bladder cancer patients – the results of electrophoresis in polyacrilamide gel under non-reducing conditions (percentage of the total amount of proteins in the serine protease fraction).

Protein fraction	Experimental groups				
	Control (*n* = 10)	Stage I (*n* = 10)	Stage II (*n* = 10)	Stage III (*n* = 10)	Stage IV (*n* = 10)
Covalent complexes of plasminogen/plasmin and their degraded forms with inhibitors, 105–220 kDa	73.20	18.82	33.56	26.22	25.33
Plasminogen/plasmin, ~95 kDa	5.17	4.84	2.26	5.77	5.62
Plasminogen/plasmin degraded forms, 45–75 kDa	16.66	68.70	59.40	65.99	64.05
Elastase, ~28 kDa	0	5.92	2.77	1.64	4.31
Trypsin, ~20 kDa	0	1.73	2.02	0.40	0.68

The percentage of plasmin and plasminogen in plasma serine protease fraction from healthy donors was 5.17%, and the amount of proteins with higher molecular weights was about 73% – they most likely were represented by plasmin/plasminogen covalent complexes as well as their degraded forms with inhibitors. Degraded forms of plasminogen were also detected in a fairly significant amount (16.7%).

Changes in the protein composition were detected in serine protease fractions obtained from plasma of BC patients, in particular, a significant decrease in the percentage of high molecular covalent complexes of plasminogen/plasmin and their degraded forms with inhibitors was noted. The relative quantity of such complexes decreased 3.8, 2.2, 2.8 and 2.9-fold in patients with BC Stages I, II, III and IV, respectively, compared to the values in the group of healthy donors.

Our results also showed that the percentage of plasminogen and plasmin in the serine protease fractions obtained from plasma of both healthy individuals and patients with all BC stages did not differ significantly and was about 5%, apart from this parameter value in patients with BC Stage II – it was 2.3 times lower compared to the control.

At the same time, a rise in the percentage of plasminogen and plasmin degraded forms was established. The value of this index was 4; 3.6; 4 and 3.8 times higher in the plasma serine protease fractions from BC patients with stage I, II, III and IV, respectively, compared to the donors group. The analysis also revealed the presence of proteins with molecular weights of 20 and 28 kDa, which might correspond to the enzymes trypsin and elastase, which were not observed in plasma from healthy donors.

Table 2 shows the results of disk electrophoresis under reducing conditions (in the presence of β-ME). The data indicated the presence of stable covalently bound complexes of proenzyme fragments with effector molecules (with a molecular weight of 80–135 kDa, 20.86%) in plasma serine protease fraction from healthy donors, while no such complexes were detected among the components of investigated fractions obtained from BC patients.

**Table 2:** Protein composition of plasma serine protease fractions from bladder cancer patients – the results of electrophoresis in polyacrilamide gel under reducing conditions (percentage of the total amount of proteins in the serine protease fraction).

Protein fraction	Experimental groups				
	Control (*n* = 10)	Stage I (*n* = 10)	Stage II (*n* = 10)	Stage III (*n* = 10)	Stage IV (*n* = 10)
Stable covalent complexes of proenzyme fragments with effector molecules, 80–135 kDa	20.86	0	0	0	0
Plasmin heavy chain, ~68 kDa	23.21	13.33	4.63	9.34	10.38
Enzymes/proenzymes fragments, 30–60 kDa	44.42	16.22	12.70	21.91	24.24
Plasmin light chain, ~25 kDa	11.51	4.42	1.91	6.68	1.20
Trypsin/protein fragments, ~19 kDa	0	8.92	23.85	17.64	29.83
(elastase and other proteins), 8–13 kDa	0	57.12	56.92	44.45	34.35

The presence of enzymes/proenzymes fragments with a molecular weight of 30–60 kDa was established in plasma serine protease fraction from healthy donors at 44.42%, while the corresponding values were only 16.2, 12.7, 21.9 and 24.2% in patients with BC Stage I, II, III and IV, respectively.

Heavy (23.2%) and light (11.5%) plasmin chains were also detected among the proteins of plasma serine protease fraction from healthy donors. The percentage of plasmin heavy chain in fractions obtained from plasma of BC patients was 13.3% (stage I), 4.6% (stage II), 9.3% (stage III) and 10.3% (stage IV), whereas the light chain levels were 4.4, 1.9, 6.7, and 1.2%, respectively.

Trypsin was not present among the components of the material obtained from plasma of healthy persons, while its percentage was 8.92, 23, 17.64 and 29.83% in plasma serine protease fractions of patients with BC Stages I, II, III and IV, resprectively.

Along with the absence of the band corresponding to the stable covalent complexes of proenzymes with effector molecules in fractions from patients with BC, the appearance of elastase fragments with molecular weight of 8–13 kDa was established, with percentagses being 57.1, 56.9, 44.5 and 34.35% for stages I – IV of BC.

## Discussion

Dysregulation of proteolysis with a rise in both protease expression and activity are signs of cancer progression (17). A tumor is a complex structure formed by cancer and stromal cells, as well as ECM. Contributing to ECM degradation, proteases participate in tumor cell invasion and metastasis, and are involved in all stages of cancer progression by the regulation of growth factor synthesis. The specific set of proteases involved in cancer progression, along with their regulators, is known as the cancer degradome, and its components are secreted by both cancer and stromal cells (15). Serine proteases, including plasmin and PAS components, are an important constituent of cancer degradome (24, 25).

Our results established that both the concentration of plasminogen and its potential activity in the plasma of BC patients exceeded that found in the healthy donors group. It was also shown that there is an increase in the serine proteases total content in plasma from patients with oncopathology compared to the control group, which is consistent with data obtained in several other studies. These enzymes is noted can be produced by both cancer and stromal cells in BC, and their overexpression usually correlates with a higher frequency of relapses and worse patient survival (8, 12, 26–29).

Additionally, our results revealed that the plasminogen concentration in patients with stage IV BC is reduced, and plasminogen potential activity significantly declined in patients with stages III – IV. Futhermore, a fall in the serine proteases concentration during BC progression was observed, which can be explained by the synthesis and (or) activation of other proteases involved in tumorigenesis which acted to reduce the concentration of the enzymes studied in this experiment. Such alterations can be results of cytokine and growth factors content modulation in the tumor microenvironment due to the change in tumor cellular compositions at the different stages of tumorigenesis. Proteolytic enzymes can also form multiprotein complexes or complexes with their inhibitors. Moreover, proteases of different classes produced by the tumor microenvironment can cross-influence each other, forming a complex and interconnected tumor proteolytic network (15).

Electrophoretic analysis demonstrated the presence of plasminogen/plasmin (95 kDa), their degraded forms (45–75 kDa), and also high-molecular covalent complexes of their native or degraded forms with inhibitors (105–220 kDa), which prevailed among the components of the studied serine protease fraction isolated from healthy donor’s plasma. Electrophoresis under reducing conditions made it possible to isolate heavy (68 kDa) and light (25 kDa) plasmin chains, as well as high-molecular stable (resistant to the reducing agents action) covalent complexes of proenzymes fragments with effector molecules (80–135 kDa) as well as the enzymes/proenzymes fragments themselves (30–60 kDa).

Plasminogen conversion into plasmin requires PAS functioning, and PAS components can be produced by both malignant and stromal cells (25, 30). Plasmin in turn proteolytically activates MMPs, which along with plasmin, are involved in the ECM degradation, promoting cancer cells invasion and metastasis formation. Futhermore, plasmin promotes tumor growth by growth factor proteolytic activation and direct stimulation of protease-activated receptors that leads to the cytokine and chemokine gene expression up-regulation, promoting cell migration and inflammation development (31, 32).

According to the electrophoretic analysis results, the percentage of plasminogen/plasmin fraction in patients with different stages of BC did not change compared to the control, with the exception of the stage II which was characterized by a decrease in plasminogen/plasmin fraction. However, a significant decline in the proportion of high-molecular covalent complexes of native and degraded forms of plasminogen and plasmin with inhibitors was revealed, with a simultaneous increase in the middle molecular weight (45–75 kDa) components content, representing degraded forms of plasminogen and plasmin. Moreover, in patients with all BC stages, trypsin and neutrophil elastase were found within the serine protease fraction components that were absent among the studied enzymes in the control group. The results of electrophoresis under reducing conditions revealed the absence of the high molecular weight fraction represented by stable covalent complexes of proenzyme fragments with effector molecules in BC patients, and also showed a significant reduction in the percentage of middle molecular weight fragments of enzymes and proenzymes, along with a decline in the heavy and light plasmin chains content. Additionally, the appearance of two new low molecular weight protein fractions was detected among the serine proteases from BC patients plasma, namely enzyme fragments with a mass of 8–13 kDa (in particular, elastase fragments), as well as trypsin and other protein fragments with a mass of about 19 kDa. An increase in the low molecular weight proteins content shown under gel electrophoresis carried out in reducing conditions, can be also partially explained by the destruction of enzymes dimeric forms stabilized by disulfide bonds, with the release of the appropriate molecular weight monomers. After all, most serine proteases undergo limited proteolysis during post-translational modification with following formation of such dimers (33).

The decline in the high-molecular weight components percentage of the plasma serine proteases fraction with a simultaneous rise in the degraded forms of plasminogen/plasmin and low-molecular weight fragments content may indicate the proteolytic processes intensification under BC development. This is confirmed by the presence of trypsin and neutrophil elastase among the studied fractions components – enzymes that were absent in healthy donors plasma.

Extrapancreatic tumor-associated trypsinogen (TAT) is produced by cancer and stromal cells under oncopathology (34). After activation it directly degrades ECM components, promoting cancer cell invasion, or acts indirectly by cleaving pro-uPA and MMPs latent forms (35).

Neutrophil elastase (NE) is contained in the granules of mature neutrophils and is released into the extracellular space during degranulation or netosis – a specific cell death type, which can be stimulated by cytokines and chemokines, generated by the tumor microenvironment, and is characterized by the extrusion of proteolytically active neutrophil extracellular traps (NETs) (36, 37). Tumor-associated neutrophils are important constituents of the tumor microenvironment, and high degree of tumor infiltration by these cells correlates with an unfavorable prognosis in BC (38–40).

NE activates a number of growth factors and cytokines and, therefore, stimulates the primary tumor growth (41). NE also activates MMPs latent forms and so is involved in ECM remodeling and angiogenesis. Additionally, as a component of the NETs, NE participates in metastasis, since NETs can sequester circulating tumor cells and contribute to their transfer along the vascular bed promoting spreading (42). Therefore, under many oncopathologies, the increased NE content correlates with the disease stage and increased mortality.

The results of electrophoresis under reducing conditions revealed a significant decline in the percentage of heavy and light plasmin chains in plasma serine proteases fractions from BC patients, which along with above mentioned alterations in plasma plasminogen content and its potential activity, may indicate certain changes in the direction of plasminogen proteolytic conversion.

It is known that plasminogen, in addition to its proteolytic conversion into plasmin, can also undergo limited proteolysis with proteolytically inactive angiostatins (ANGs) formation that are counted to be angiogenesis and cellular migration inhibitors. And NE is considered to be an enzyme that can alter the plasminogen transformation direction. In this way, NE competes for the substrate with tPA and uPA, depleting the plasminogen circulating pool, which leads to the inability to generate a sufficient amount of plasmin (43). Futhermore, ANGs may repress tPA activity as well as competitively displace plasminogen from the complexes with its receptors, preventing this proenzyme conversion into plasmin (44). Therefore, NE catalytic activity inhibits fibrinolysis under tumor progression, which increases the cancer-associated thrombosis risk. This is consistent with a number of studies that indicate a causal relationship between NETs formation, NE activity, and hypercoagulation (45, 46).

## Conclusion

The study of the proteolytic activity and distribution of serine proteases in plasma from BC patients is relevant, as these enzymes are involved in tumor growth, angiogenesis, and metastasis in the setting of malignancy. It was established that the concentration of plasminogen, its potential activity as well as the total amount of serine proteases in plasma from BC patients exceeded the values of the corresponding indicators in healthy donors which indicates an increase in proteolysis as a contribuiting factor to carcinogenesis. However, the plasminogen concentration in BC patients with stage IV disease demonstrated a reduction compared to healthy controls, and plasminogen relative activity significantly declined in patients with stages III – IV. Futhermore, a gradual decline in the serine proteases plasma content was shown in BC patients depending on the tumor stage. The results of electrophoretic analysis established a significant reduction of high molecular weight components content (under non-reducing conditions) and their complete absence (under reducing conditions) in plasma serine proteases fractions from BC patients. Additionally, a rise in the degraded forms of plasminogen/plasmin content was shown, and the presence of fractions absent in the control, which correspond to trypsin and NE (under non-reducing conditions), and other low-molecular weight fragments (under reducing conditions). Therefore, the changes of the cancer degradome were observed under tumor progression. It can be assumed that NE appearance in BC patients plasma is a consequence of tumor infiltration by immune cells and the generation of pro-inflammatory cytokines and chemokines in the tumor microenvironment, which stimulate netosis and NETs releasing into circulation, which become a source of elastase. In turn, NE switches the plasminogen proteolysis into direction of ANGs formation. This can explain both the decline in the relative activity of plasminogen (that is, essentially plasmin) in plasma from patients with latter stages of BC, and the corresponding trend found for the plasma plasminogen levels (due to NE-dependent cleavage of this protein), as well as a decrease in the percentage of heavy and light plasmin chains in serine proteases fractions obtained from BC patients plasma (because plasmin is not formed in the usual amount). Therefore, our results in combination with previous literature reveal a number of the proteolysis features in patients with various stages of BC and allow proposing the possible mechanisms of their emergence.

As only men were included in the study group, the obtained results can be interpreted only in relation to men with BC and can not be applied to women as well as to the general cohort of patients with this malignancy (without taking into account gender).

## Data availability statement

The original contributions presented in the study are included in the article/supplementary material, further inquiries can be directed to the corresponding authors.

## Ethics statement

The studies involving humans were approved by the Ethics Committee at the ESC “Institute of Biology and Medicine,” Kyiv, Ukraine (the protocol No 9, 13.11.2020). The studies were conducted in accordance with the local legislation and institutional requirements. The participants provided their written informed consent to participate in this study.

## Author contributions

TS: Data curation, Investigation, Validation, Writing – original draft, Writing – review & editing. TV: Data curation, Funding acquisition, Investigation, Validation, Writing – original draft. TH: Formal analysis, Investigation, Methodology, Visualization, Writing – review & editing. VT: Participated in statistical data processing. NR: Data curation, Funding acquisition, Investigation, Validation, Writing – review & editing. OS: Conceptualization, Formal analysis, Resources, Supervision, Writing – review & editing. TF: Formal analysis, Investigation, Resources, Validation, Writing – original draft. LO: Conceptualization, Funding acquisition, Methodology, Project administration, Writing – review & editing. PY: Conceptualization, Investigation, Methodology, Project administration, Resources, Software, Writing – original draft, Writing – review & editing. MK: Formal analysis, Methodology, Software, Visualization, Writing – review & editing. DT: Conceptualization, Methodology, Project administration, Resources, Writing – original draft. KS: Funding acquisition, Investigation, Methodology, Project administration, Software, Visualization, Writing – original draft, Writing – review & editing.
